# Overcoming Target Driven Fratricide for T Cell Therapy

**DOI:** 10.3389/fimmu.2018.02940

**Published:** 2018-12-12

**Authors:** Eytan Breman, Benjamin Demoulin, Sophie Agaugué, Sebastien Mauën, Alexandre Michaux, Lorraine Springuel, Julien Houssa, Fanny Huberty, Céline Jacques-Hespel, Céline Marchand, Jérôme Marijsse, Thuy Nguyen, Nancy Ramelot, Benjamin Violle, Dorothée Daro, Peter De Waele, David E. Gilham, Valérie Steenwinckel

**Affiliations:** Celyad S.A., Mont-Saint-Guibert, Belgium

**Keywords:** chimeric antigen receptor, T cells, PI3K inhibitor, NKG2D, CD314, NKG2D blocking antibody, fratricide

## Abstract

Chimeric Antigen Receptor (CAR) T cells expressing the fusion of the NKG2D protein with CD3ζ (NKG2D-CAR T Cells) acquire a specificity for stress-induced ligands expressed on hematological and solid cancers. However, these stress ligands are also transiently expressed by activated T cells implying that NKG2D-based T cells may undergo self-killing (fratricide) during cell manufacturing or during the freeze thaw cycle prior to infusion in patients. To avoid target-driven fratricide and enable the production of NKG2D-CAR T cells for clinical application, two distinct approaches were investigated. The first focused upon the inclusion of a Phosphoinositol-3-Kinase inhibitor (LY294002) into the production process. A second strategy involved the inclusion of antibody blockade of NKG2D itself. Both processes impacted T cell fratricide, albeit at different levels with the antibody process being the most effective in terms of cell yield. While both approaches generated comparable NKG2D-CAR T cells, there were subtle differences, for example in differentiation status, that were fine-tuned through the phasing of the inhibitor and antibody during culture in order to generate a highly potent NKG2D-CAR T cell product. By means of targeted inhibition of NKG2D expression or generic inhibition of enzyme function, target-driven CAR T fratricide can be overcome. These strategies have been incorporated into on-going clinical trials to enable a highly efficient and reproducible manufacturing process for NKG2D-CAR T cells.

## Introduction

Improvements in our understanding of the immune system have led to the development of many immune-focused approaches that are now delivering objective clinical responses in patients with advanced cancer. One of these approaches is the Chimeric Antigen Receptor (CAR) T cell where patients' T cells are gene-modified to express a tumor targeting CAR and then returned to the patient in large numbers (1). Through the impressive outcomes seen in CD19 antigen bearing B cell malignancies, this adoptive cell therapy has now been added to the validated therapeutic approaches in those cancers. This is now driving exploration beyond the initial successes to address non B cell hematological cancers as well as solid tumors (2–7). The CAR concept is also moving beyond T cells with CARs being explored in a range of cell types including Natural Killer (NK) cells (8).

CARs are modular protein receptors that consist of a target binding domain linked to structural regions that generally include an extracellular spacer domain and a transmembrane region fused to intracellular signaling domains. Upon ligand binding, downstream signaling initiated from the CAR activates the effector function of the T cell, driving direct tumor cell killing and immune-recruiting cytokine production.

T cells engrafted with a CAR comprising the human Natural Killer Group 2 D (NKG2D) sequence fused with the cytoplasmic domain of CD3ζ undergo effector activation upon engagement with target ligand (9). Importantly, the NKG2D-CD3ζ fusion protein is stabilized at the T cell surface by the interaction with the adaptor protein DAP-10 that upon NKG2D ligand binding, initiates a co-stimulatory signal much like that of CD28. Thus, this molecular complex acts like a second-generation CAR producing ligand-induced activatory and co-stimulatory signals that drive full T cell effector activation.

NKG2D has eight known ligands including the major histocompatibility complex class I- related A and B proteins (MICA and MICB, respectively) and the UL16–binding protein (ULBP) family (ULBP1-6) (10). Expression of these NKG2D ligands is known to be induced under “stress” conditions such as cellular damage, infection, oxidative or thermal stress, or malignant transformation. As a result, NKG2D ligands are found to be broadly expressed across a majority of hematological and solid tumors (11).

Consequently, a CAR based upon NKG2D enables the targeting of both hematological and solid cancer through the use of a single CAR construction (12). This concept has been extensively demonstrated in numerous pre-clinical syngeneic mouse models where mouse T cells bearing the murine NKG2D CAR effectively eradicate a range of established hematological and solid tumors (13–18). Human T cells bearing the human NKG2D-CAR T cells engage effector cell function against a range of tumor cells *in vitro* and can similarly challenge established human tumors in xenograft mouse model (19).

Initial clinical testing of NKG2D-CAR T cells involved the infusion of a very small dose of freshly prepared CAR T cells to patients with advanced hematological malignancies [CM-CS1 trial (20, 21)] in order to establish an early safety profile. The next step of clinical testing of NKG2D-CAR (termed NKR-2 from here on) T cells requires significant up-scaling and cryopreservation to deliver the required dosing schedule for the THINK clinical trial (NCT03018405). The THINK trial continues to test NKR-2 T cells in patients with advanced hematological malignancy and adds testing in patients with solid tumors including colorectal and ovarian carcinomas (22). Unlike all other CART trials, the THINK clinical study explores the safety profile and initial activity indications in a stand-alone approach (i.e., without standard preconditioning) and tests a paradigm of multiple infusions. The initial preclinical work yielded very promising outcomes with a multiple infusion scheme, and, if positive, this approach could greatly enhance the safety profile of CAR T therapies (22).

Since multiple injections were required, significant upscaling of cell manufacture and CAR T cell cryopreservation were needed to enable the reliable production of the required dose for each patient from a single apheresis. Initially, both up-scaling and cryopreservation of NKR-2 T cells resulted in poor cell yields, hypothetically due to self-killing or fratricide. T cell fratricide is well-understood as a mechanism to maintain T cell homeostasis (23); however, in the therapeutic setting, T cell fratricide prevents the ability to produce the desired number of T cells for clinical applications. This is particularly pertinent in the situation where the target itself is chosen for T cell lineage specificity such as CD7 (24) or CD5 (25) to enable targeting of T cell leukemias. However, the issue is not restricted to T cell therapy. T cells armed with high affinity transgenic T cell receptors specific for survivin (BIRC5) undergo fratricide due to expression of the target antigen (26, 27).

For NKR-2 T cells, target driven fratricide was strongly hypothesized as the reason for the loss of cell viability. Two different strategies based upon either an inhibitor or an antibody were explored as means to control fratricide. Interestingly, both approaches-controlled fratricide to differing levels with a hybrid of both approaches producing a method that reproducibly generated NKR-2 T cells suitable to deliver the necessary doses to treat patients at all dose levels in the THINK clinical trial. Moreover, these findings have broader applicability for T cell therapies where fratricide is an issue.

## Materials and Methods

### Antibodies and Flow Cytometry

Cells were stained with fluorochrome labeled CD3 (BD, 345766), CD4 (BD, 345809), CD8 (BD, 345772), CD314 (BD, 558071), CD45RA (BD, 550855), CD62L (BD, 555544), CD279 (eBioscience, 12-2799-42), CD19 (BD, 345791, CD223 (eBioscience, 25-2239-41), MICA/B (R&D Systems, FAB13001G-100), MICB (R&D Systems, FAB1599G), ULBP1 (R&D Systems, FAB1380C), ULBP2/5/6 (R&D Systems, FAB1298A), ULBP3 (R&D Systems, FAB1517P), ULBP4 (R&D Systems: FAB6285A), and corresponding isotypes according to standard protocols. Briefly, cells were harvested and resuspended in a buffer containing DPBS (Life Technologies, A1285801) supplemented with 5% human serum albumin (Octapharma, 68209-633-02) and 0.01% NaN_3_ (Sigma, S2002). Cells were incubated with antibodies for 30min at 4°C, washed with PBS and analyzed on a Guava easyCyte 6HT cytometer (Millipore). Antibodies were all titrated prior to experimental use. Viable cells were selected based on FSC/SSC. In all instances, an unlabeled control and an isotype control were used. Analysis was performed using FlowJo v10.

### Plasmids and Vector Production

Chimeric NKG2D (chNKG2D) construct was made as previously described (13) and cloned into the Mo-MLV-based oncoretroviral vector SFG between NcoI and XhoI restriction sites. The pSFG GFP plasmid and pSFG htCD19.1 [coding for a truncated form of human CD19 (tCD19)] were a kind gift from Celdara Medical LLC (Lebanon, New Hampshire, USA). GP2-293 packaging cells were transiently transfected with the relevant plasmid together with VSV-G envelop plasmid. Retrovirus suspensions produced in PG2-293 cells were used to spin-transduce PG13 cells to obtain stable producer cell lines. Vector particles used to transduce human T lymphocytes were harvested from PG13 stable producer cells after the culture reached confluence. Vector titers were measured using the Retro-X^TM^ qRT-PCR Titration Kit (Life Technologies, CL 631453).

### NKG2D-CAR T Cell Production

Peripheral blood mononuclear cells (PBMC) were isolated from whole blood of healthy donors (ImmuneHealth, CHU, Tivoli) by ficoll density gradient (VWR, 17-5442-03) according to standard procedures. Briefly, whole blood was diluted three times with DPBS and added carefully onto the ficoll layer in a 50 ml tube. Tubes were centrifuged at 500 g and the intermediate layer carefully removed. The PBMCs were subsequently washed three times with DPBS, harvested and subsequently activated in *X-Vivo* 15 medium (Westburg, BE02-061Q) containing 5% of human sera (Access Biologicals, 515-HI) and supplemented with 40 ng/ml OKT3 (Miltenyi, 170-076-124) and 100 IU/ml IL-2 (Miltenyi, 170-076-146). Cells were incubated for 2 days in an incubator maintained at 37°C, 5% CO_2_. Cells were subsequently harvested and transduced in 24-well (1 × 10^6^ cells/well) plates coated with 8 μg/ml retronectin (Life Technologies, T100B) with different viral vectors and incubated for 2 days. Cells were then harvested from the 24 well plates, washed with HBSS (Westburg, BE10-543F) and transferred to G-Rex containers (Wilson Wolf, 80040S) for the expansion phase for an additional 4 days in complete *X-Vivo* 15 containing serum and IL-2 or as described in the text. At the end of the expansion phase, cells were harvested and used accordingly.

### Cell Lines and Culture Reagents

The chronic myeloid leukemia cancer cell line K562 and the pancreatic cancer cell line PANC-1 were purchased from ATCC and maintained in IMDM (Westburg, LO BE12-722F) or DMEM (Westburg, LO BE12-604F), respectively, containing 10% FBS (Gibco, 16140071) and 1% penicillin streptomycin (ThermoFisher Scientific, 15140122) until the time of use. The PI3K inhibitor LY294002 was purchased from Selleck Chemicals (S1105). Inhibiting antibodies to NKG2D or corresponding isotypes were purchased from BioLegend (Inhibiting antibody (Ultra-leaf CD314) clone: 1D11, ImTec Diagnostics NV, 320814).

### Cytolytic Assay

Adherent PANC-1 cells were cultured for 20 h in a flat-bottom 96-well plate in the presence or absence of thawed NKR-2 T cells or tCD19 transduced cells at a ratio of 1:1 in *X-Vivo* 15 without phenol red containing 5% human sera (HS). T cells were washed and remaining adhering PANC-1 cells were labeled for 4 h with alamarBlue (ThermoFisher Scientific, DAL1025). Viable cells were measured using Fluorescence at 530 nm using a SpectraMax M2 (Molecular Devices) and the relative cytolytic activity calculated.

### Cytokine Release Assay

Fresh NKR-2 T cells and/or control tCD19 cells were incubated with K562 or PANC-1 cells at a ratio of 1:1 in *X-Vivo* 15 containing 5% HS. Following an incubation of 24h supernatants were harvested and IFN-γ measured by ELISA (R&D Systems, SIF50) according to manufacturer's protocol. As a positive control cells were activated with PMA (Sigma-Aldrich, P8139-5MG) and ionomycin (Sigma-Aldrich, I0634-1MG). To assess the background levels of activation the cells received no stimulus.

### Antibody Inhibition Assay

NKR-2 T cells were incubated with 1 μg/mL of NKG2D blocking antibody, isotype control or no antibody for 24 h and subsequently NKR-2 T cells mediated secretion of IFN-γ measured. Similarly, NKR-2 cells were co-cultured with cancer cells in the presence of antibody and cytokine secretion measured.

### RNA Extraction and qPCR

PBMCs were stimulated with 40 ng/mL OKT3 and IL-2 (100IU/mL) for 2 days, transduced and cultured for two more days with 40 ng/mL OKT3 and IL-2 (100IU/mL), then expanded in the presence of IL-2 (100 IU/mL) until day 8 or 10 as described in detail in the text. Total RNA was isolated every 2 days using the RNeasy Mini Kit (Qiagen, 74104). Quantitative PCR reactions were conducted using pre-designed TaqMan gene expression assays (Hs04187752_mH, Hs01026642_m1, Hs00607609_mH, Hs00906262_m1, Hs00741286_m1, Hs01584111_mH, Hs04194671_s1, Hs00360941_m1, ThermoFisher Scientific) for the NKG2D ligands and Light Cycler 480RNA master mix (Roche, 04991885001). Relative expression was based on the housekeeping gene cyclophilin using in house designed primers (5′-GACGGCGAGCCCTTGG-3′ and 5′-GCACGAAAATTTTCTGCTGTCTT-3′) and probe (5′ TEX615-TCTCCTTTGAGCTGTTTGCAGACAAGGT-3′ BHQ™). Results are presented as fold induction compared to day 0 (calculated as 2^∧−^ΔΔCT). All gene expression assays were tested on different cancer cell lines known to express the ligands.

### Statistical Analysis

Where applicable an unpaired, paired two tailed *t*-test or non-parametric Mann–Whitney *U*-test, was used to assess statistical significance. Statistical significance was considered when *p* < 0.05.

## Results

### NKR-2 T Cells Undergo Target-Driven Fratricide That Drives the Phenotype and Expansion of the Engineered T Cell Population.

**Figure 1:**
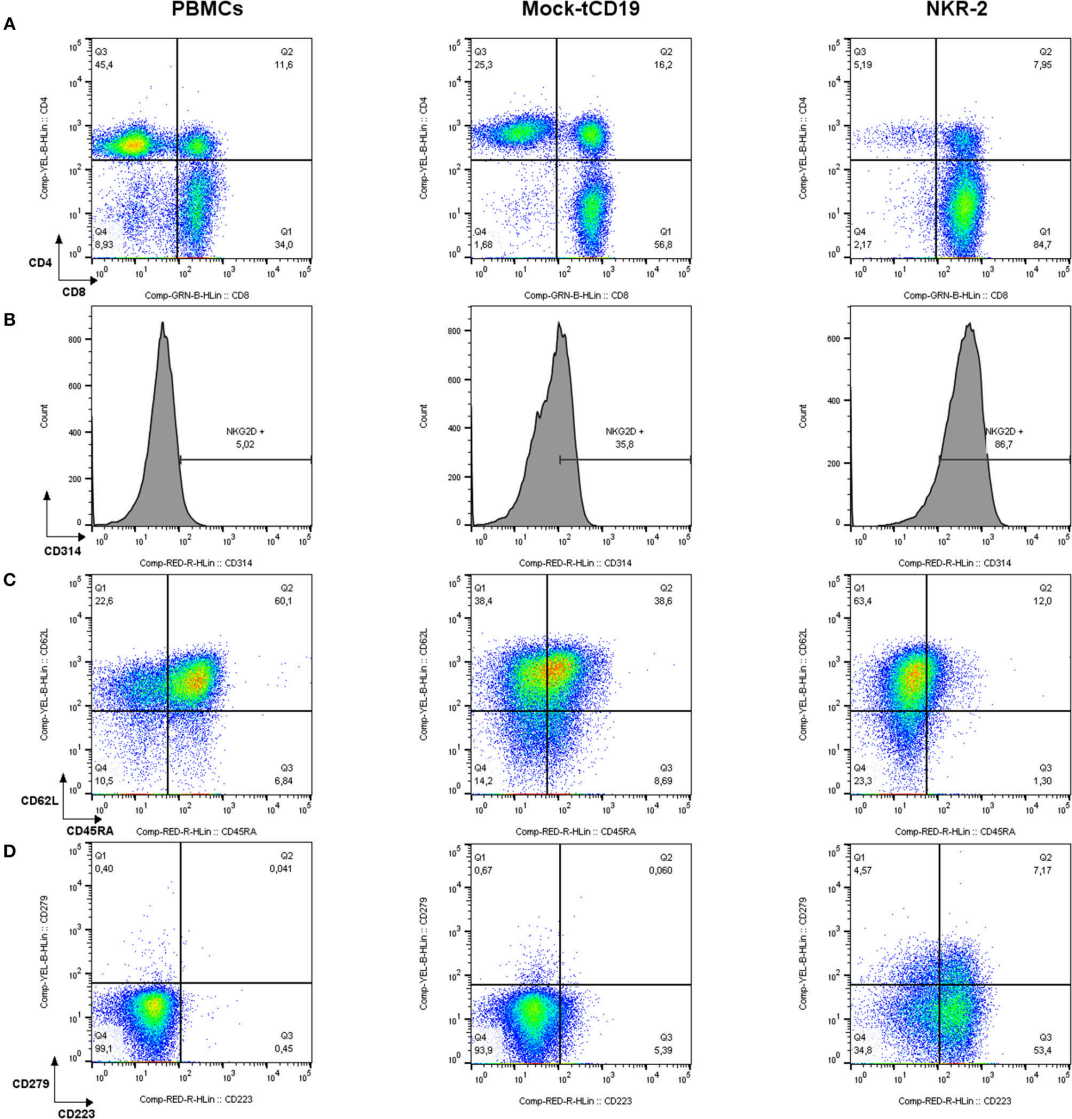
Flow cytometric characterization of PBMCs, tCD19, and NKG2D-CAR T cells in a representative NKR-2 T cells process. Mononuclear cells purified from blood using density gradient method were analyzed before (PBMCs) and after (tCD19 and NKR-2 T cells) the NKR-2 T cells process. After acquisition, cells were gated on SSC/FSC for Lymphocytes. Lymphocytes were then gated on CD3. All CD3 positive cells were then displayed as followed: **(A)** CD4/CD8 distribution. **(B)** Surface expression of NKG2D (CD314). **(C)** Memory phenotype. CD62L and CD45RA for the distinction of Naïve (CD62L+ CD45RA+), central memory (CD62L+ CD45RA–), effector memory (CD62L– CD45RA–), and terminal differentiated T cells expressing CD45RA (CD62L– CD45RA+) and **(D)** Exhaustion phenotype as stained for CD223 (Lag-3) and CD279 (PD-1). One representative donor out of 3 is shown.

After transduction and *in vitro* culture, in the absence of methods to control fratricide, NKR-2 T cell populations display a predominantly CD8^+^ T cell subset composition as compared to T cells transduced with a control vector (truncated CD19 (tCD19), Figure 1A). NKG2D expression is not exclusive to NKR-2 T cells but also clearly visible on control tCD19 T cells although engagement of the endogenous NKG2D fails to deliver a therapeutic response in the CAR T cell (28) However, the relative cell surface expression of NKG2D was highly increased in the NKR-2 T cell population indicative of transduction of the T cells with the CAR construct (Figure 1B). The mean fluorescence intensity of NKG2D (CD314) was significantly higher in the NKR-2 T cell populations in both CD4^+^ and CD8^+^ subsets confirming expression of the CAR in both subsets (Supplement Figures 1A,B and Supplement Table 1).

**Figure 2:**
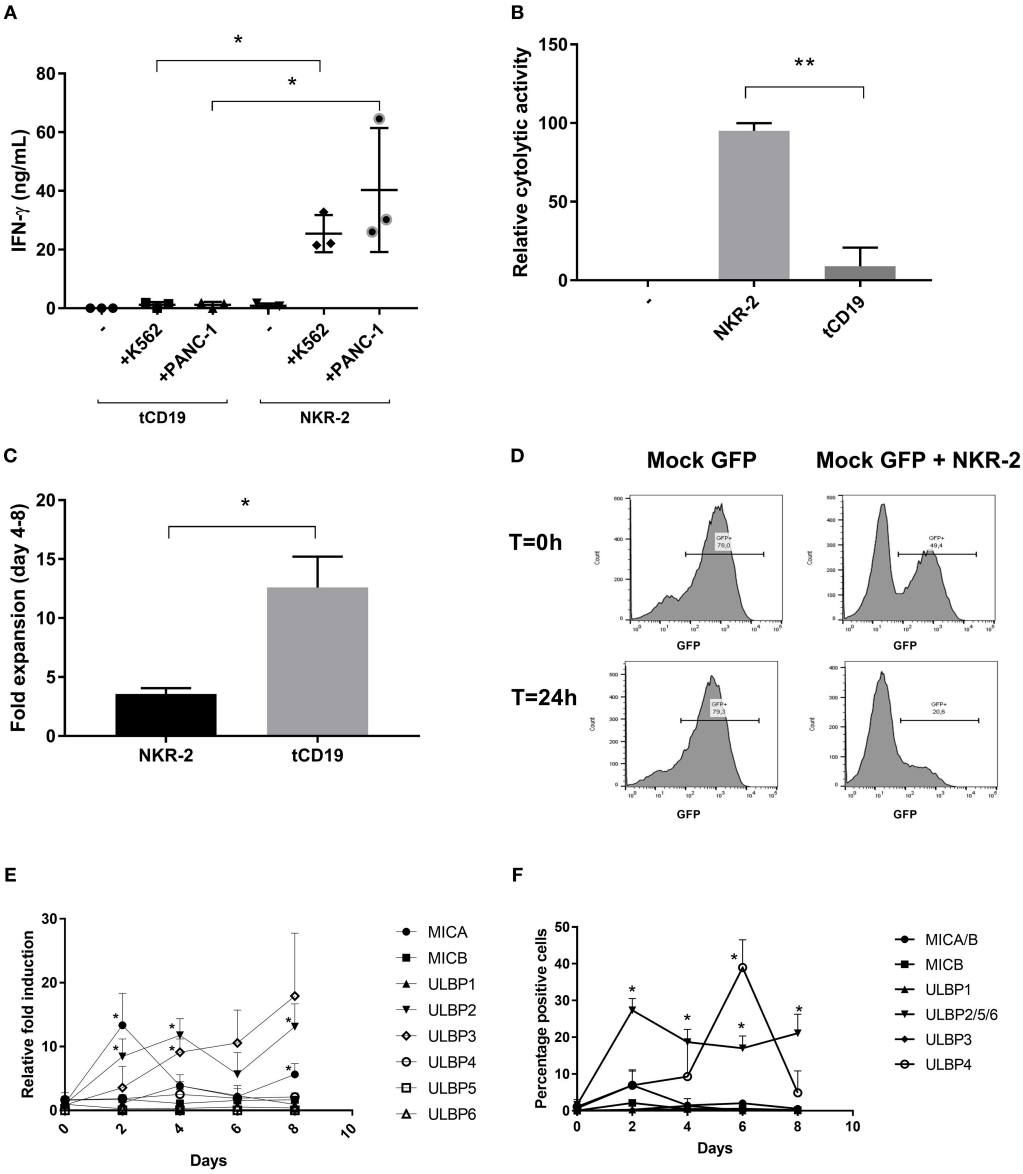
NKR-2 T cells recognize Chronic myeloid leukemia (K562) and Pancreatic Carcinoma (PANC-1) but display a fratricide effect due to NKG2DL expression. **(A)** T cells from healthy donors were transduced with tCD19 vector (tCD19 T cells) or with the NKR-2 T cells vector (NKG2D-CAR T cells) and co-cultured with indicated cell lines or alone (–). IFN-γ secretion (ng/ml) was quantified by ELISA after overnight coculture. Each data point represents the mean value of duplicate wells from independent experiments. Shown is (*N* = 3). **(B)** NKR-2 T cells exhibit cytolytic activity. PANC-1 were cocultured with thawed NKG2D-CAR T cells at an E:T ratio of 1:1. Alamar blue signal was determined after 20 h. Percent lysis was determined by absorbance comparison with untreated cancer cells (PANC-1). Data shown are mean ± SD of *N* = 3 independent T cell donors.; –, PANC-1 cells alone; NKR-2 T cells: Coculture of NKR-2 T cells and PANC-1 cells; tCD19, Coculture of control tCD19 T cells with PANC-1 cells. **(C)** Transduced T cells were cultured in complete *X-vivo* (100 IU/mL IL-2) for 4 days. T cells were analyzed 96 h after seeding to analyze fold expansion relative to the initial cell seeding density. Data shown are mean ± SD of *N* = 3 independent T cell donors. **(D)** Representative co-culture experiment (out of three). GFP positive T cells were either cultured alone (Mock-GFP) or co-cultured with NKR-2 T cells generated from the same donor (Mock GFP + NKR-2 T cells). Flow cytometry analysis of GFP positivity was acquired at the beginning of the incubation (*T* = 0 h) or after 24 h of incubation (*T* = 24 h). **(E,F)** PBMCs were activated with 40 ng/mL anti-CD3 and 100 IU/mL IL-2 and kept in culture for a total of 8 days in accordance with the normal manufacturing protocol. Every 2 days cell samples were harvested and the expression of NKG2D ligands analyzed as either RNA **(E)** or proteins on the cell surface **(F)**. A two-tailed unpaired *t*-test was used to assess statistical significance. A *p* < 0.05 was considered significant (*) and *p* < 0.01 (**). For both qPCR and Flow cytometric comparison a paired two-tailed *t*-test was used.

Interestingly, the NKR-2 T cell population displayed a reduced relative frequency of naïve cells (as defined by CD45RA^+^ and CD62L^+^ cells double positive) and increased frequency of CD279 (PD-1) and CD223 (Lag-3) when compared to the tCD19 control T cell population suggestive of an increased effector cell phenotype (Figures 1C,D). NKR-2 T cells demonstrated high levels of target cell induced IFN-γ secretion (Figure 2A) and cytolytic activity (Figure 2B) upon co-culture with cancer cell lines confirming the functionality of NKR-2 T cells. However, the yield/fold expansion (Figure 2C) and viability (data not shown) of NKR-2 T cells during the culture and upon harvest was consistently reduced as compared to the tCD19 control T cells.

NKG2D ligand expression has been documented on T cells during mitogenic activation (29) while it is known that NK cells can undergo fratricide as a consequence of NKG2D receptor engagement (30, 31). Together, this raised the question whether NKR-2 T cells were undergoing fratricide after transduction putatively driving low cell yield and viability, CD4/CD8 ratio bias and enhanced effector memory differentiation. To test this, T cells from a donor were transduced with an eGFP expression vector and mixed with NKR-2 T cells generated from the same donor to explore whether general T cell killing was occurring. Twenty-four hours later, there was a clear loss of eGFP T cells in the NKR-2 T cells co-culture implying targeted killing of autologous T cells by NKR-2 T cells (Figure 2D).

To understand the NKG2D ligand expression profile in activated T cells, three healthy donors were used as a source of T cells for activation without transduction and qPCR analysis was performed to examine the kinetics of NKG2D ligand mRNA expression profile (Figure 2E). A rapid increase in MICA and ULBP2 mRNA levels was detected within 2 days of T cell activation. However, while the level of ULBP2 mRNA remained high, MICA was quickly reduced to the baseline within a further 2 days. The mRNA for ULBP3 increased gradually over the time-course while there was no relative increase in transcripts for MICB, ULBP5, ULBP1 nor ULBP6. Conversely, ULBP4 mRNA was slightly increased at day 4 but then reduced again to basal level. At the cell surface protein level, MICA/B followed a similar expression pattern to the corresponding mRNA, with a transient presence of MICA on day 2, that subsequently diminished (Figure 2F). Unfortunately, no suitable antibodies were available to detect the individual ULBP2/5 and 6 proteins; however, the immune-reactivity observed with the antibody that recognizes all three family members was most likely due to ULBP2 based upon mRNA expression profile, which was the only one increased after day 2. Whilst highly induced at the mRNA level, no cell surface ULBP3 could be detected. ULBP4^+^ cells reached a peak of positivity by day 6 before dropping back to baseline by day 8 following the pattern of mRNA expression with a two days delay (Figure 2F). These observations were reflected in a parallel kinetics of mean fluorescence intensity (Supplemental Table 2).

Together, these data imply that T cells modulate expression of NKG2D ligands after mitogenic activation with MICA, ULBP4, and putatively ULBP2 being the NKG2D ligands that predominate at the protein level albeit with differing kinetics of expression.

### PI3K Inhibition Improves NKR-2 T Cell Viability Upon Cryopreservation, but Does Not Improve the Yields of NKR-2 T Cells

Upon ligand engagement, NKG2D and the associated DAP10 protein initiate signal transduction through the PI3K pathway in a manner similar to that of CD28 (32, 33). Therefore, we questioned whether inhibition of PI3K signaling could abrogate NKR-2 T cells mediated fratricide during T cell culture. To this end, increasing concentrations of LY294002 were added during the transduction and expansion phases of NKR-2 T cells culture.

**Figure 3:**
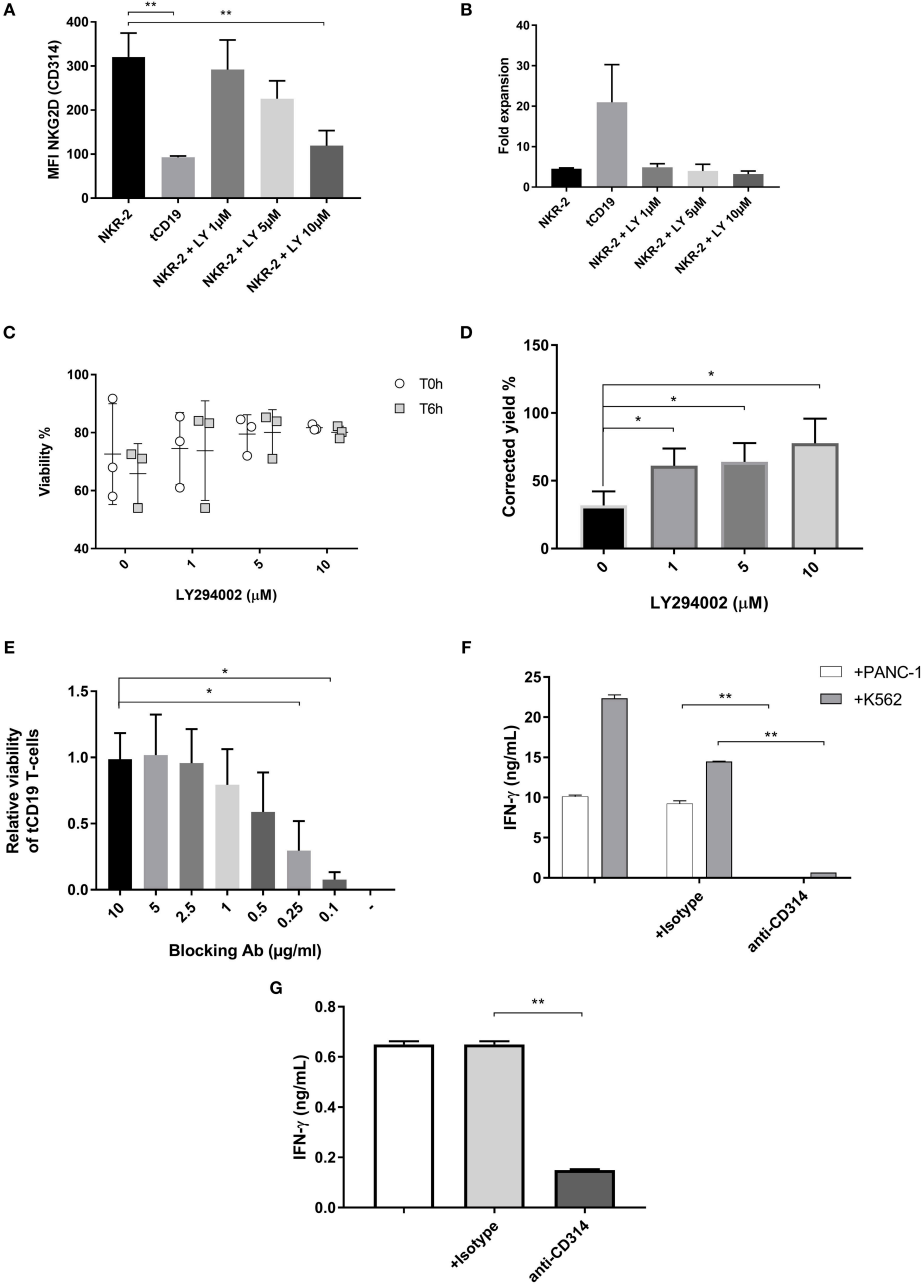
NKR-2 T cells fratricide is modulated by a PI3K inhibitor or a blocking antibody. **(A)** MFI of NKG2D expression on NKR-2 T cells treated or not with increasing concentrations of LY294002. Data shown are mean ± SD of *N* = 3 independent donors. **(B)** Transduced T cells were cultured in complete *X-vivo* (100 IU/ml IL-2) supplemented or not with increasing concentrations of LY294002 for expansion. T cells were analyzed 96 h after seeding for fold expansion relative to the initial cell seeding density. Data shown are mean ± SD of *N* = 3 independent donors. **(C)** Viability of cells after cryopreservation. NKR-2 T cells were produced in presence of increasing concentration of LY294002. After production, cells were harvested, washed, and formulate for cryopreservation. After cryopreservation, cells were thawed using a water bath and resuspended in Plasmalyte/Human Serum Albumin (HSA) 5%. Cells viability was directly assessed after thawing (T0h) or after 6 h at 4°C in Plasmalyte/HSA5% (T6h) (*N* = 3) **(D)** NKR-2 T cells were produced in presence of increasing concentration of LY294002. After production, cells were harvested, washed, transferred in Plasmalyte/HSA1% (50 × 10^6^ NKR-2 T cells/ml) and stored at 4°C for 48 h. After 48 h, cell viability was assessed using trypan blue staining (*N* = 3) and normalized for the number of cells at cryopreservation **(E)** tCD19 T cells were co-cultured with NKR-2 T cells generated from the same donor in presence of increasing concentrations of blocking Ab [from 0 (–) to 10 μg/ml]. Flow cytometry analysis of CD19 positivity and viability was acquired after 44 h of incubation. Data are normalized with CD19 positivity of Mock cultured without NKR-2 T cells (*N* = 3). **(F)** Thawed NKR-2 T cells were cocultured with either PANC-1 cells or K562 cells at a 1:1 ratio, in the presence of CD314 blocking Ab, an isotype control or no Ab. Following a 24 h incubation, supernatants were harvested and IFN-γ measured (*N* = 3). **(G)** Thawed NKR-2 T cells were left in culture for 24h in the presence of an isotype control, the CD314 blocking Ab (or no Ab) and IFN-γ levels measured. Data shown are mean ± SD of *N* = 3 independent donors. A two-tailed unpaired *t*-test was used to assess statistical significance. A *p* < 0.05 was considered significant (*) and **p < 0.01.

The addition of LY294002 resulted in several observations. Firstly, the cell surface expression of NKG2D on NKR-2 T cells was reduced in a dose-dependent manner, reaching the level of control tCD19 T cells at 10 μM (Figure 3A and Supplement Figure 2). This reduction was furthermore reversible as removal of LY from the culture led to an increase in NKG2D expression up to the levels of untreated NKR-2 T cells (data not shown). However, there was no discernable improvement in cell yield with the inhibitor suggesting that fratricide during culture was either not being managed completely or that the PI3K inhibitor had a deleterious effect on proliferation (Figure 3B). To assess whether LY294002 had an anti-proliferative effect, control tCD19 T cells were treated with the PI3K inhibitor during the culture. A clear reduction in the proliferative capacity of these control T cells was observed when compared to untreated tCD19 cells (Supplement Figure 3).

As expected, NKR-2 T cells manufactured with PI3K inhibitor showed an increase in cell viability both after cryopreservation or when stored at 4°C for 48 h (Figures 3C,D). NKR-2 T cells produced using PI3K inhibitor produced greater quantities of IFN-γ in an LY294002 dose-dependent manner (Supplement Figure 4A). Finally, NKR-2 T cells cultured with LY294002 also appeared to have an increased CD62L^+^/CD45RA^−^ phenotype (Supplement Figure 4B) in agreement with previously published work using this inhibitor to modulate the memory phenotype of T cells (34).

Overall, the addition of the PI3K inhibitor had beneficial effects upon NKR-2 T cells viability but cell yields remained poor due to either the impact on T cell proliferation or the remaining NKG2D expressed on the cell surface.

### Antibody Mediated NKG2D Blockading Prevents NKR-2 T Cell Fratricide

An initial experiment that included an anti-NKG2D antibody (clone 1D11) during the culture of NKR-2 T cells showed that NKR-2 T cell yield at the end of culture was equivalent to that of control T cells (2.6-fold expansion for NKR-2 T cells vs. 13.8-fold expansion for NKR-2 T cells with antibody blockade (Supplement Figure 5). This suggested that antibody blockade could abrogate NKG2D target-driven fratricide. A dose-titration experiment showed that antibody concentrations of 2.5 μg/mL and above resulted in protection of tCD19 T cells from NKR-2 T cell targeted killing (Figure 3E). The anti-NKG2D antibody also effectively blocked IFN-γ release completely during target cell engagement (Figure 3F) thereby confirming the specificity of the NKR-2 T cells. The effective blocking of fratricide using the anti-NKG2D antibody was further supported by the fact that the IFN-γ release observed during the NKR-2 T cell production, putatively due to T cell fratricide, was significantly reduced by the addition of the blocking antibody (Figure 3G). Since the mouse blocking antibody could potentially cause toxicities by antibody-dependent cell-mediated cytotoxicity (ADCC), extensive washing steps were realized after harvest. IgG ELISA and flow cytometry experiments indicated that no contaminating antibody could be detected in the supernatant or on the cell surface after harvest (Data not shown). To assess ADCC, cocultures of NK cells with autologous NKR-2 cells in the presence of 5 μg/mL of Ab were conducted with no evidence of ADCC (data not shown). Together these data suggest that the addition of anti-NKG2D blocking antibody controlled NKR-2 T cells CAR driven fratricide.

### Adaptation of the Blocking Ab Process During *in vitro* NKR-2 T Cell Expansion Allows for Increased Yields With Equivalent Activity

**Figure 4:**
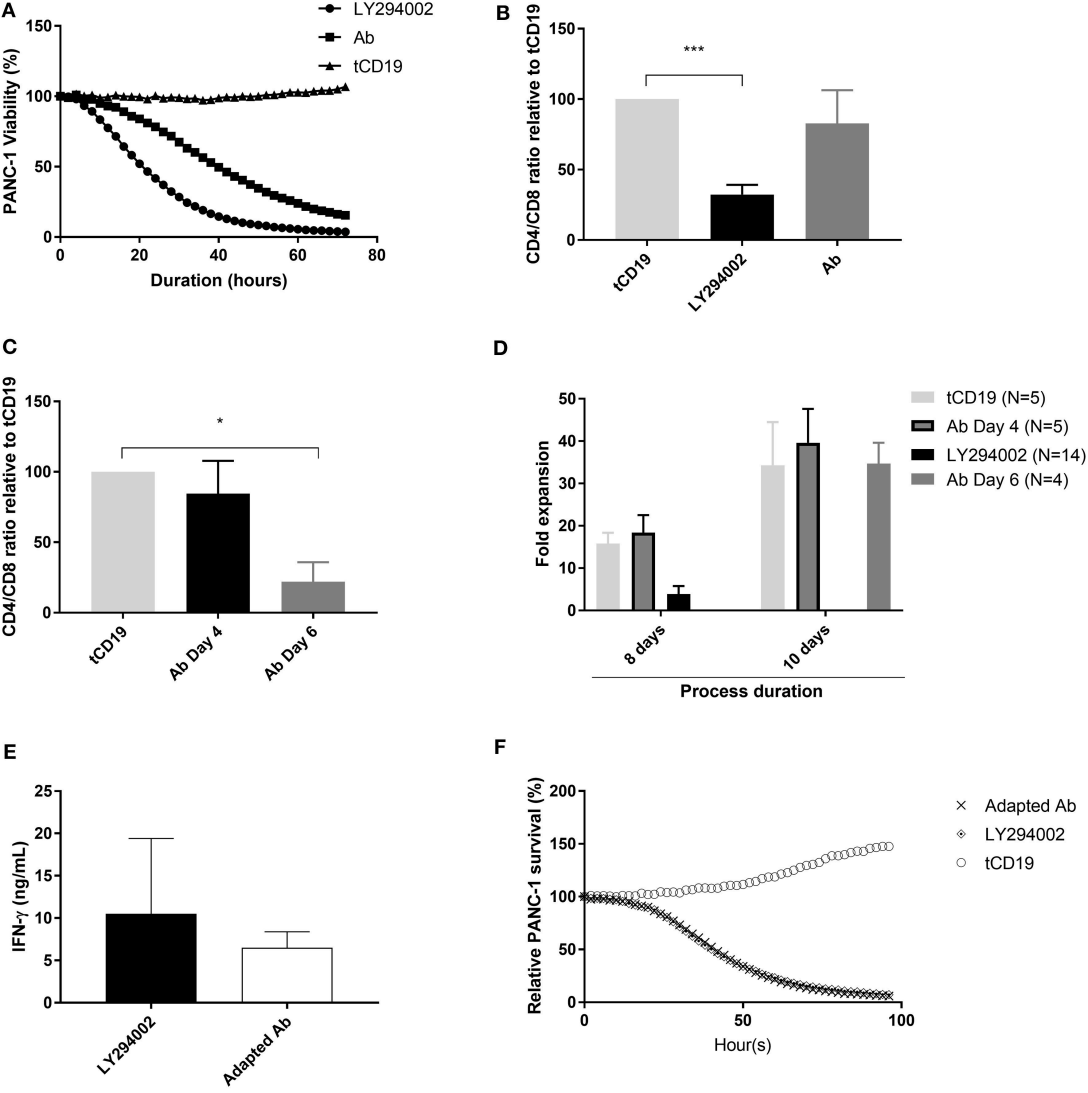
NKR-2 T cells Ab blockade process adaptation restores CD4/CD8 ratio**. (A)** Cytolytic activity kinetics, one representative killing assay out of three. Thawed control tCD19 T cells or NKR-2 T cells treated with PI3K inhibitor LY294002 or with the blocking Ab were cultured in presence of NucLight positive PANC-1. PANC-1 viability was assessed every 2 h by the IncuCyte S3 device. **(B)** CD4/CD8 distribution at harvest. During expansion phase, NKR-2 T cells were either cultured with 5 μM of LY294002 or with 5 μg/mL of blocking Ab for 96 h, harvested and measured by Flow cytometry for the CD4 and CD8 population. Data shown are mean ± SD of *N* = 4 independent T cell donors relative to the control tCD19 ratio. **(C)** CD4/CD8 distribution at harvest with a delayed addition of blocking Ab. During expansion phase (day 4 to day 8), NKR-2 T cells were either directly treated with 5 μM of blocking Ab (day 4) or after 48h (day 6). At harvest (day8), T cells were harvested and analyzed by Flow cytometry for the CD4 and CD8 populations. Data shown are mean ± SD of *N* = 3 independent T cell donors relative to control tCD19 CD4/CD8 ratio. **(D)** Comparison of expansion. NKR-2 T cells were either cultured for 8 or 10 days in presence of LY or blocking Ab (added at day 4 or at day 6). T cells were analyzed at harvest for fold expansion relative to the initial cell seeding density. **(E)** Potency assay by IFN-γ secretion. Thawed NKR-2 T cells processed by the two methods were cocultured in presence of PANC-1 cells. IFN-γ secretion was measured by ELISA after 44 h of co-culture. Data shown are mean ± SD of *N* = 4 independent T cell donors. **(F)** Cytolytic activity kinetics, one representative killing assay out of three. Thawed control tCD19 T cells or NKR-2 T cells treated with PI3Ki or with the blocking antibody were cultured in presence of NucLight positive PANC-1 cells. PANC-1 viability was assessed every 2 h by the IncuCyte S3 device. A two-tailed unpaired *t*-test was used to assess statistical significance. A *p* < 0.05 was considered significant *p < 0.01 and ***p < 0.001.

A comparison between NKR-2 T cells produced using the antibody and PI3K inhibitor processes showed different cytolytic kinetics, implying a change in T cell characteristics with the Ab process (Figure 4A). In comparing the two processes the main difference observed was the CD4/CD8 ratio that, on day 8, was consistently skewed toward a CD8 population when the PI3K inhibitor was added. Interestingly, blocking of NKR-2 T cells rescued the CD4^+^ population, suggesting that the skewed CD4/CD8 ratio observed is fratricide dependent (Figure 4B). The most likely explanation for the difference in ratio is either a relative increase in the ratio due to proliferation of the CD4 T cells, or a removal of the CD4 T cells by the CD8 T cells.

We hypothesized that the augmented lytic activity of NKR-2 T cells produced with the PI3K inhibitor could be due to the lower CD4/CD8 ratio. To address this, we adapted the blocking antibody process by adding the blocking antibody on day 6 instead of immediately following transduction (day 4). This modification led to the production of NKR-2 T cells exhibiting a CD4/CD8 ratio similar to that of the cells produced with the PI3K inhibitor (Figure 4C), while maintaining a fold expansion comparable to control T cells (Figure 4D). Subsequently, despite minor differences in certain parameters between the two processes such as the expression of the activation marker CD25 and the memory phenotype (Supplement Table 3), functional cytokine secretion and cytolytic activity of NKR-2 T cells against target cancer cells was comparable between both processes (Figures 4E,F).

In a preliminary *in vivo* experiment using NOD SCID gamma mice receiving LY and Ab process generated NKR-2 cells showed a similar anti-tumor activity in an established acute myeloid leukemia (THP-1) tumor model (8 days post infusion as visualized by bioluminescence; tCD19: 5.76E10 ± 4.46E10; NKR-2 LY: 7.15E08 ± 1.01E09; NKR-2 Ab: 6.43E08 ± 1.25E08; data not shown). A one way ANOVA with Tukey's *post-hoc* test showed significant differences between LY and tCD19 control cells (*p* = 0.02) and Ab produced NKR-2 compared to tCD19 (*p* = 0.03). No difference was observed between LY and Ab groups (*p* = 0.95). Additionally, a similar engraftment after 24 h was observed in both NKR-2 LY and Ab group (LY group: 1.838 ± 1.07%; Ab group: 1.792 ± 0.56%; data not shown). Indicating that no significant difference could be detected in short-term engraftment between the two groups.

Taken together, these combined data indicated that NKR-2 T cells produced with the adapted blocking antibody process exhibited a similar short-term engraftment and potency to the cells produced using the PI3K inhibitor.

## Discussion

The recent approval of CD19 T cell therapy in B cell acute lymphoblastic leukemia (bALL) and diffuse large B cell lymphoma (DLBCL) provides a strong clinical validation of CAR-T therapy and provides an impetus to develop T cell therapy beyond CD19^+^ B malignancies. The choice of target is essential to the success of the therapy, yet the identification of tumor exclusive antigens has been challenging. A recent bioinformatics study combining proteomic and genomic approaches in AML, showed that there was no tumor specific cell surface antigen, and that complex combinatorial targeting strategies may be required for antibody-based T approaches to target AML (35). Consequently, most target antigens being tested at present are tumor associated antigens where expression of the target may also be present on normal, healthy cells. There are many examples including CD19 for B cell malignancies (5, 6), CD123 in AML (36), CD7 (24) and carcinoembryonic antigen (CEA) in a range of solid tumors (37).However, in the case where the target antigen may be permanently or transiently expressed on a T cell, this leads to T cell fratricide, which practically translates into a reduced cell yield.

Gene editing now provides a clinically relevant method to prevent the expression of specific proteins thereby enabling the expansion of T cells that would otherwise undergo fratricide (24). However, the multi-target specificity of the NKG2D based CAR means that gene editing to eliminate eight different proteins in the primary T cell along with efficiently expressing the CAR construct is currently beyond the practical capability of clinically-relevant gene editing technology. Thus, other strategies are required to avoid fratricide occurring during cell culture to enable the production and delivery of a NKG2D-focused T cell therapy.

In this study, a PI3K inhibitor provided a generic approach to control fratricide by reducing the NKG2D expression on the cell surface. This is, to our knowledge, the first time that such observation is reported. The underlying mechanism responsible for the loss of cell surface NKG2D after PI3K inhibitor treatment is currently unknown. Given that NKG2D cell surface localization is mediated by its association with DAP10 (33), one hypothesis could be that DAP10 is impacted by the PI3K inhibitor treatment [e.g., decreasing RNA levels, inhibiting transcription or inhibition of post-translational modifications such as glycosylation, which are required for the association of DAP10 with NKG2D (38)]. This could ultimately result in the prevention of expression of the NKG2D-DAP10 complex on the cell surface and inhibition of fratricide. However, one major consequence in inhibiting the PI3K pathway is its association with reduced cell proliferation (39–42), rendering this approach limited when high number of cells are required for clinical application.

An alternative approach to inhibit fratricide during the manufacturing of NKR-2 T cells, was the use of a specific blocking antibody during the expansion phase. This enabled the control of T cell fratricide and the expansion of T cells to levels equivalent to that of control tCD19 T cells. This method is strongly dependent on the antibody used, as it needs to block fratricide without inducing CAR activation itself. The addition of the specific blocking antibody provides a solution that enables the large-scale expansion of NKR-2 T cells.

Taken together, these data demonstrate that T cell fratricide can be managed by generic methods such as PI3K inhibition or by receptor-specific approaches such as a blocking antibody. Each offers potential advantages that can be used to generate T cell products when self-expression of the target ligand is a limiting factor, where other means such as gene-editing to eliminate the target in the T cell population are not currently feasible in the clinical situation.

## Ethics Statement

This study was carried out in accordance with the recommendations of the VOXCAN ethical committee (CEAA-129). This protocol was approved by the VOXCAN ethical committee. This project was also submitted for review to the French authorities (Ministry of National Education, the Higher Education and Research) and was approved.

## Author Contributions

EB and BD designed the study conducted experiments analyzed results and wrote the manuscript. SA and SM discussed results and reviewed the manuscript. AM and LS preformed experiments and analyzed results. JH, FH, CJ-H, CM, JM, TN, NR, and BV preformed experiments. DD designed and analyzed the incucyte experiments. PD helped design the study and reviewed the article. VS designed the study discussed results and reviewed the article. DG discussed results and wrote the manuscript.

### Conflict of Interest Statement

All authors involved in the manuscript were employees of Celyad SA. The research conducted within the manuscript may lead to the development of products which may be licensed to Celyad SA. The reviewer MM and handling editor declared their shared affiliation.

## Abbreviations

CAR: Chimeric Antigen Receptor
NKG2D: Natural Killer Group 2 D
AML: Acute Myeloid Leukemia
Ab: Antibody.
