# Supplementary material for: Overcoming Target Driven Fratricide for T Cell Therapy
Source: Front Immunol. 2018 Dec 12;9:2940. doi: 10.3389/fimmu.2018.02940 (PMC6299907; doi:10.3389/fimmu.2018.02940)
Supplement: Supplementary file 1 [file Table_1.docx]

**Supplements:**

**Supplement Table 1: MFI of CD314+ cells**

| **Condition** | **N** | Mean Fluorescence Intensity ± SEM | | | | | |
| --- | --- | --- | --- | --- | --- | --- | --- |
|  |  | **CD3^+^CD4^+^CD314^+^** | | | **CD3^+^CD8^+^CD314^+^** | | |
| Non-transduced | 9 | 17 | ± | 1 | 83 | ± | 7 |
| Mock tCD19 | 11 | 23 | ± | 1 | 87 | ± | 4 |
| NKR2 | 16 | 110 | ± | 14 | 298 | ± | 21 |

**Supplement Table 2: MFI of NKG2D ligand expression on CD3+ T cells**

| **Day** | **Mean Fluorescence Intensity ± SD** | | | | | | | | | | | | | | | | | |
| --- | --- | --- | --- | --- | --- | --- | --- | --- | --- | --- | --- | --- | --- | --- | --- | --- | --- | --- |
|  | **MICA/MICB** | | | **MICB** | | | **ULBP1** | | | **ULBP2/5/6** | | | **ULBP3** | | | **ULBP4** | | |
| 0 | 17.5 | ± | 0.8 | 8.4 | ± | 1.2 | 11.9 | ± | 0.5 | 15.9 | ± | 1.8 | 18.0 | ± | 0.1 | 19.0 | ± | 0.4 |
| 2 | 22.0 | ± | 4.8 | 13.5 | ± | 0.5 | 14.4 | ± | 0.3 | 24.7 | ± | 0.8 | 21.6 | ± | 1.2 | 29.7 | ± | 6.0 |
| 4 | 24.9 | ± | 2.9 | 14.9 | ± | 1.5 | 15.8 | ± | 1.2 | 28.6 | ± | 2.5 | 24.3 | ± | 1.5 | 36.9 | ± | 13.6 |
| 6 | 27.5 | ± | 1.2 | 12.8 | ± | 2.2 | 14.9 | ± | 0.2 | 27.8 | ± | 1.6 | 28.1 | ± | 1.3 | 80.3 | ± | 16.9 |
| 8 | 25.0 | ± | 1.4 | 14.9 | ± | 0.4 | 15.8 | ± | 0.3 | 28.1 | ± | 1.8 | 24.6 | ± | 0.7 | 28.9 | ± | 2.0 |

**Supplement Table 3: Characteristics of cells generated with LY294002 Vs Adapted Ab NKR-2 T Cells processes**

| **Parameters** | **LY294002 (n=4) Mean±SD** | **Ab (n=4) Mean±SD** |
| --- | --- | --- |
|  |  |  |
| Viability (%) | 90.4±7.4 | 97.3±1.9 |
| Vector Copy Number | 2.0±0.19 | 2.1±0.25 |
| CD3^+^/CD4^+^ (%) | 11.3±4.7 | 9.6±4.1 |
| CD3^+^/CD8^+^ (%) | 78.8±4.6 | 82.9±4.6 |
| Interferon gamma release (ng/mL) | 10.5±8.9 | 6.5±1.9 |
| Time to achieve 50% killing (hours) | 36.8±6.1 | 37.0±4.7 |
| Maximum killing at 96 hours (%) | 94.0±1.8 | 95.3±1.0 |
| CD3^+^ CD223 (LAG3)^+^ | 26.8±8.4 | 12.2±6.1 |
| CD3^+^ CD279 (PD-1)^+^ | 0.4±0.1 | 0.6±0.3 |
| CD3^+^/CD45RA^+^/CD62L^-^ | 3.1±1.2 | 8.7±4.3 |
| CD3^+^/CD45RA^-^/CD62L^+^ | 33.9±5.3 | 21.1±7.4 |
| CD3^+^/CD25^-^/CD69^+^ | 0.2±0.2 | 3.3±1.4 |
| CD3^+^/CD25^+^/CD69^-^ | 88.9±3.1 | 42.2±15.3 |

**Supplement legends:**

**Supplemental Figure 1**: Mean Fluorescence Intensity (MFI) of CD314. (**A-B)** MFI of CD314 in CD3^+^CD4^+^ or in CD3^+^CD8^+^ T cells upon harvest of non-transduced (N=9), Control tCD19 transduced (N=11) or NKG2D-CAR transduced T cells (N=16). Significance was tested using Welch’s t test (**** P<0.0001)

**Supplemental Figure 2:** Representative histogram of CD8+ NKG2D positivity at harvest. T-cells were gated on SSC/FSC; CD3+; CD8+; NKG2D histogram. A “positivity” cut-off is set at 95% of the PBMCs at day 0 (dashed line). Next to the mean fluorescent intensity of NKG2D the Vector Copy number (VCN) is shown.

**Supplemental Figure 3:** Fold expansion between days 4-8 of control tCD19 T cells cultured in the presence or absence of PI3K inhibitor LY294002. PBMCs from seven different healthy donors were activated for two days and at day two transduced with tCD19 expressing control vectors either in the presence or absence of 5µM LY294002. Cells were harvested at day 8 and the fold expansion between days 4 and 8 was calculated for the two conditions. Each bar represents the mean and SD of n=7. A two-tailed unpaired t-test was used to assess statistical significance. A p<0.05 was considered significant (*).

**Supplemental Figure 4: (A)** NKR-2 T cells treated with increasing concentrations of LY294002 during the process were co-cultured with indicated cell lines. IFN-γ secretion (ng/mL) was quantified by ELISA after overnight coculture. Each data point represents the mean value of duplicate wells. One representative from three independent experiments is shown. (**B)** One representative flow cytometry dot plot showing memory phenotype. NKR-2 T cells or control tCD19 T cells, treated or not with LY294002 (10µM) and stained with anti-CD62L and -CD45RA (N=3). A two-tailed unpaired t-test was used to assess statistical significance. A p<0.05 was considered significant (*), p<0.01 (**) and p<0.001 (***).

**Supplemental Figure 5**: Impact of blocking Ab on NKR-2 T cell expansion. NKR-2 T cells were either cultured without treatment (NKR-2) or with Ab from day 2 until day 8 (NKR-2 T cells + Ab) and fold expansion compared to Mock. Fold expansion is relative to the initial cell seeding density. N=1.
