# Supplementary figures and images for: Overcoming Target Driven Fratricide for T Cell Therapy
Source: Front Immunol. 2018 Dec 12;9:2940. doi: 10.3389/fimmu.2018.02940 (PMC6299907; doi:10.3389/fimmu.2018.02940)

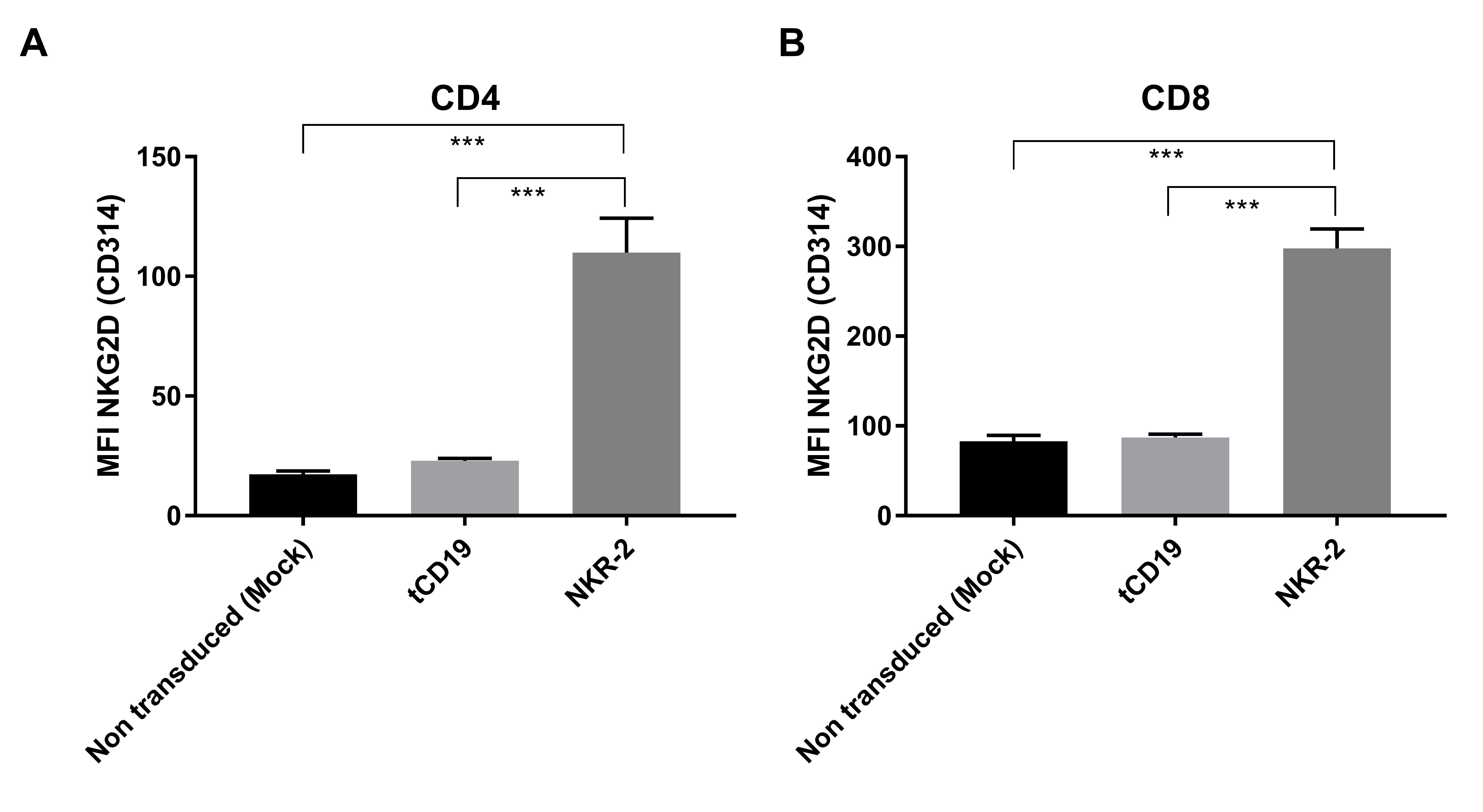

Supplement: Supplementary file 2 [file Image_1.TIF]

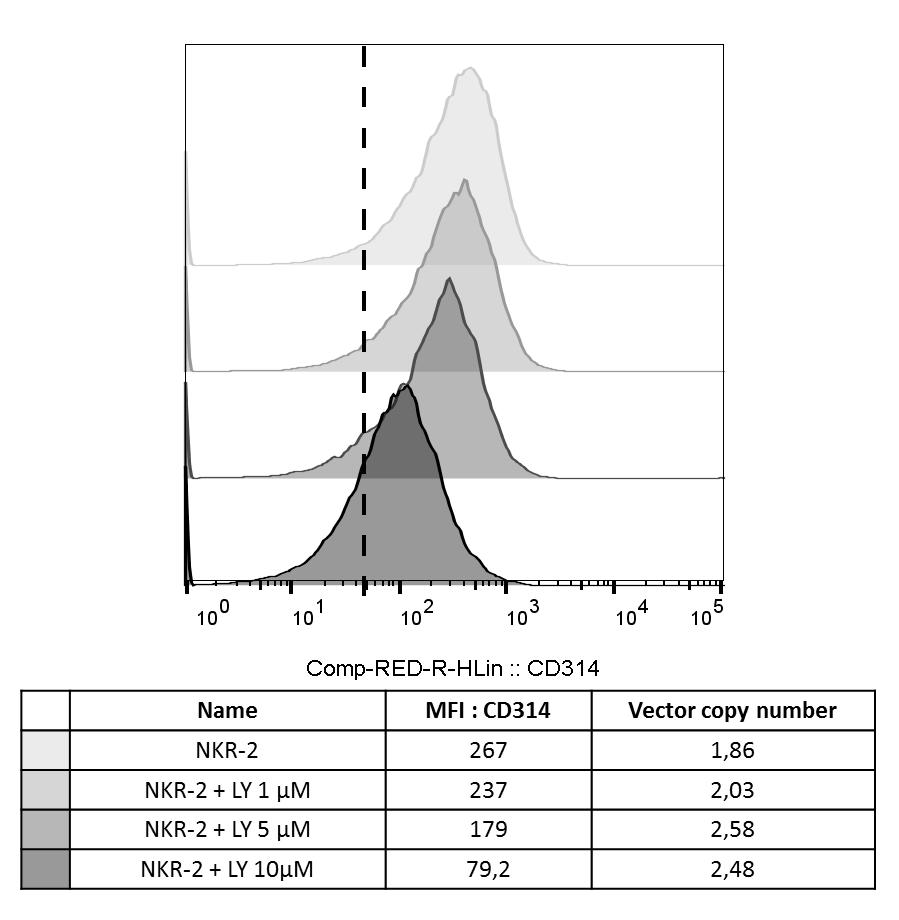

Supplement: Supplementary file 3 [file Image_2.TIFF]

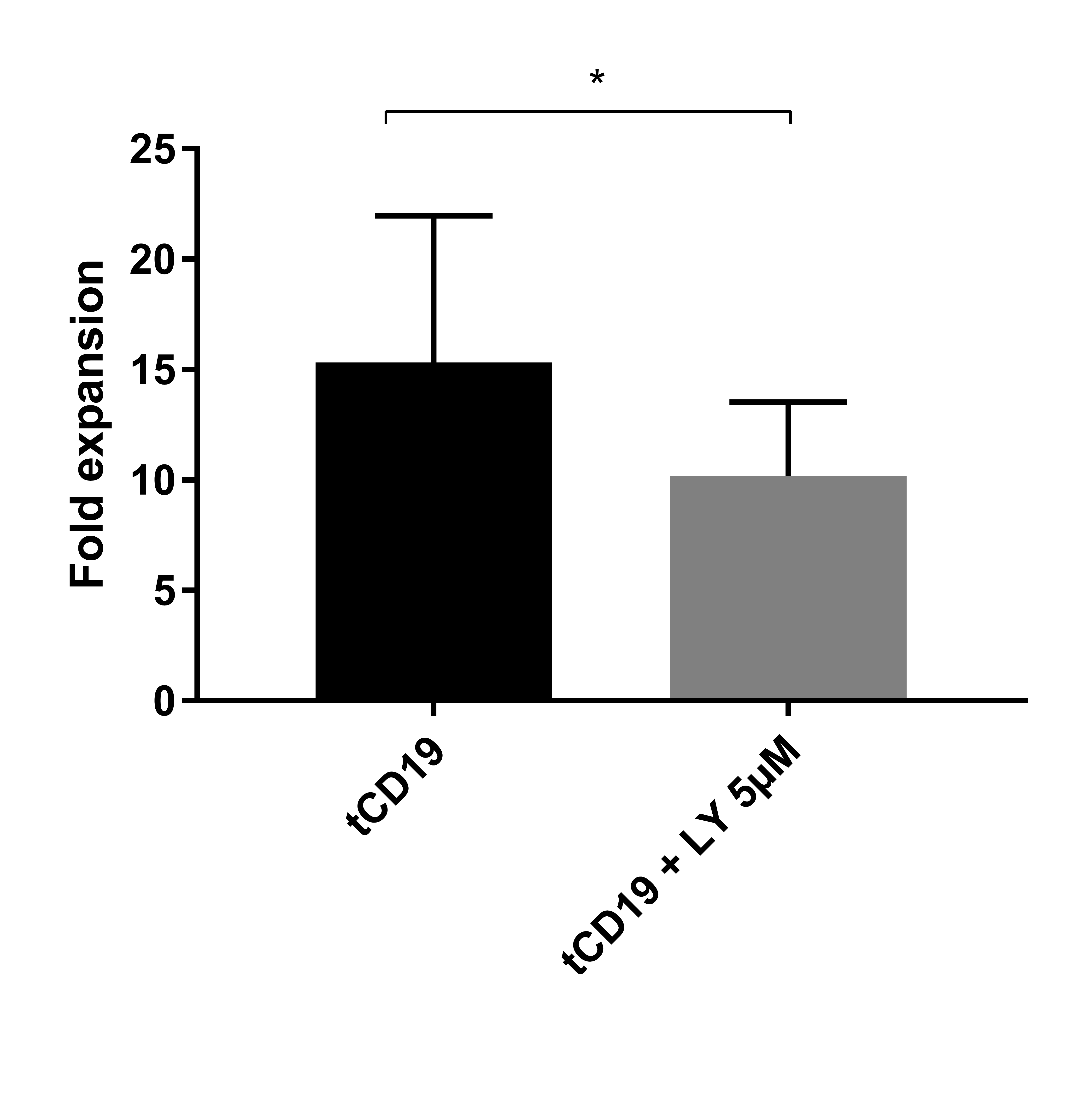

Supplement: Supplementary file 4 [file Image_3.TIF]

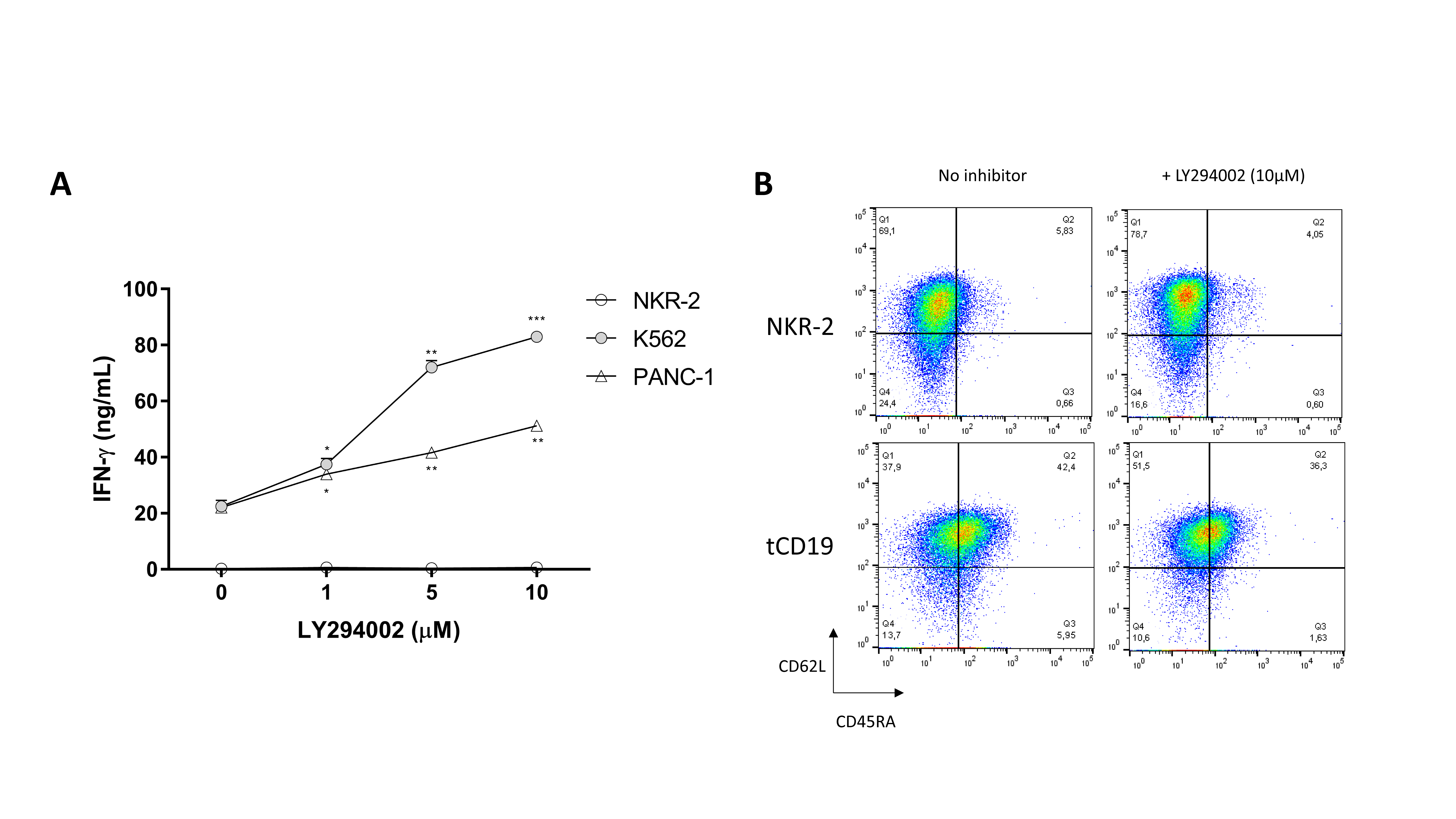

Supplement: Supplementary file 5 [file Image_4.TIF]

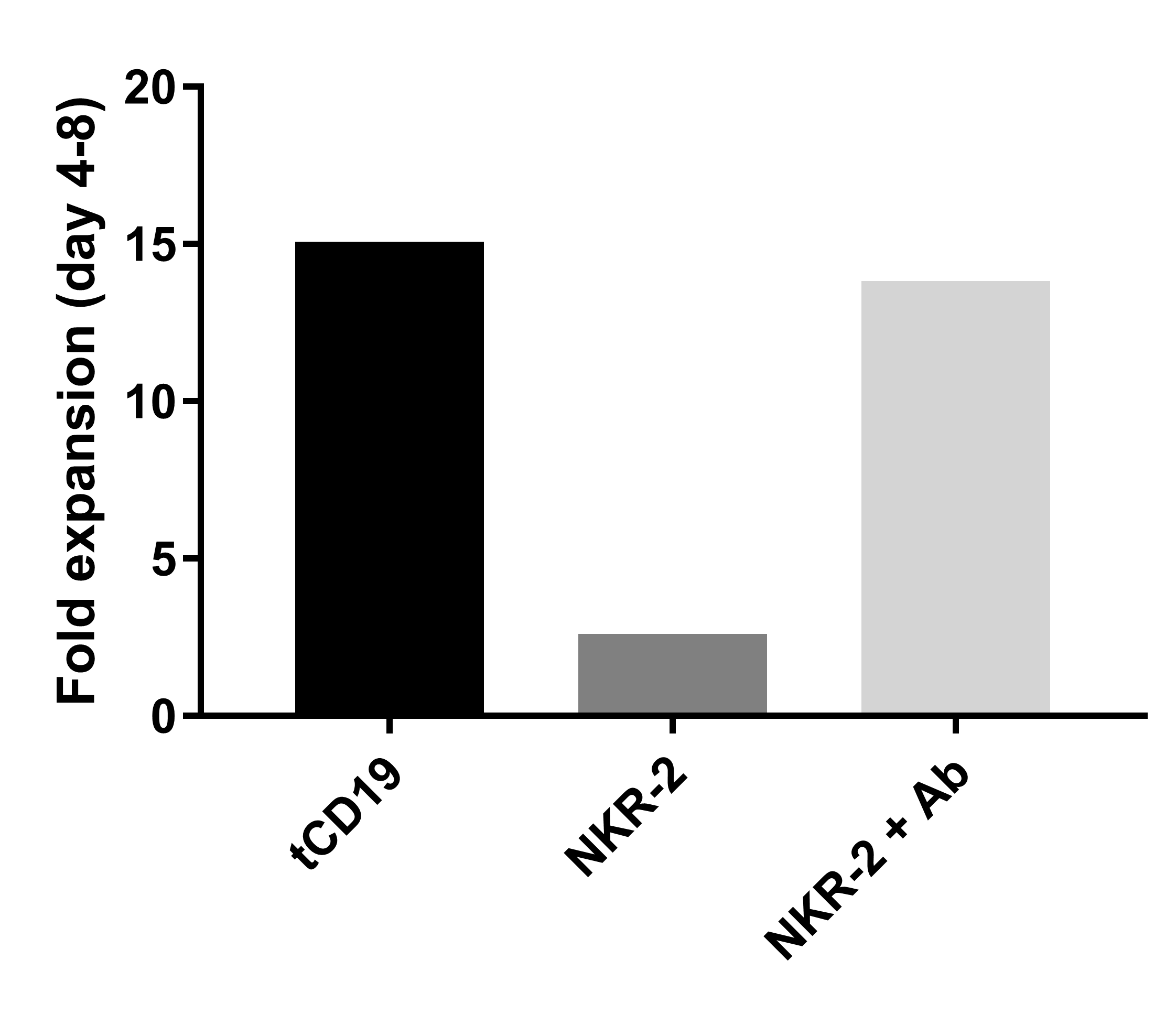

Supplement: Supplementary file 6 [file Image_5.TIF]
